# Implementation of a group physical therapy program for Veterans with knee osteoarthritis

**DOI:** 10.1186/s12891-020-3079-x

**Published:** 2020-02-03

**Authors:** Kelli D. Allen, Brandon Sheets, Dennis Bongiorni, Ashley Choate, Cynthia J. Coffman, Helen Hoenig, Kim Huffman, Elizabeth P. Mahanna, Eugene Z. Oddone, Courtney Van Houtven, Virginia Wang, Sandra Woolson, Susan N. Hastings

**Affiliations:** 1 Center of Innovation to Accelerate Discovery and Practice Transformation, Durham VA Medical Center, 508 Fulton St, Durham, NC 27705 USA; 2 Department of Medicine and Thurston Arthritis Research Center, University of North Carolina, Chapel Hill, North Carolina USA; 3 Physical Medicine and Rehabilitation Service, Durham VA Medical Center, Durham, USA; 4 Department of Biostatistics and Bioinformatics, Duke University, Durham, USA; 5 Center for Aging and Human Development, Duke University, Durham, USA; 6 Department of Medicine, Duke University Medical Center, Durham, USA; 7 Department of Population Health Sciences, Duke University Medical Center, Durham, USA; 8 Geriatrics Research Education and Clinical Center, Durham VA Health Care System, Durham, USA; 9 Center for the Study of Aging, Duke University School of Medicine, Durham, USA

**Keywords:** Osteoarthritis, Physical therapy, Quality improvement

## Abstract

**Background:**

A previous randomized clinical trial found that a Group Physical Therapy (PT) program for knee osteoarthritis yielded similar improvements in pain and function compared with traditional individual PT. Based on these findings the Group PT program was implemented in a Department of Veterans Affairs Health Care System. The objective of this study was to evaluate implementation metrics and changes in patient-level measures following implementation of the Group PT program.

**Methods:**

This was a one-year prospective observational study. The Group PT program involved 6 weekly sessions. Implementation metrics included numbers of referrals and completed sessions. Patient-level measures were collected at the first and last PT sessions and included the Western Ontario and McMaster Universities Osteoarthritis Index (WOMAC; self-report of pain, stiffness and function (range 0–96)) and a 30-s chair rise test.

**Results:**

During the evaluation period, 152 patients were referred, 80 had an initial session scheduled, 71 completed at least one session and 49 completed at least 5 sessions. The mean number of completed appointments per patient was 4.1. Among patients completing baseline and follow-up measures, WOMAC scores (*n* = 33) improved from 56.8 (SD = 15.8) to 46.9 (SD = 14.0); number of chair rises (*n* = 38) completed in 30 s increased from 10.4 (SD = 5.1) to 11.9 (SD = 5.0).

**Conclusions:**

Patients completing the Group PT program in this implementation phase showed clinically relevant improvements comparable to those observed in the previous clinical trial that compared group and individual PT for knee osteoarthritis. These results are important because Group PT can improve efficiency and access compared with individual PT. However, there were some limitations with respect to attendance and completion rates, and program adaptations may be needed to optimize these implementation metrics. Larger, longer-term studies are required to more fully evaluate the effectiveness of this program.

## Background

Physical therapy (PT) is a key component of treatment for knee osteoarthritis (OA) [1], with particular benefits for addressing deficits in strength, flexibility, function and gait. Physical therapists can play a critical role in providing instruction in an appropriate home exercise program, which is also essential in OA management [1]. Studies suggest that in the U.S., PT is underutilized for managing OA. For example, in one large U.S. database study, only 26% of individuals with OA received physical therapy over a 5-year period [2]. In analyses of participants in three different clinical trials of interventions for knee OA, at baseline only about half of participants reported that they had ever received PT for knee OA [3]. Reasons for low use of PT in this patient group are not well understood. However, efforts to enhance the efficiency of PT care delivery models for common conditions like OA are important, particularly given the projected shortage of physical therapists nationally [4] and the specific challenges to PT access in rural and other underserved settings.

Group-based PT is one delivery model that can improve efficiency, and it also offers the advantage of peer support. The Good Life with osteoArthritis in Denmark (GLA:D™) program is an example of a group session-based model of physical therapist-delivered exercise and education for osteoarthritis [5]. GLA:D™ participation has been associated with clinically relevant improvements in pain and function and demonstrated feasibility for dissemination across four countries, providing excellent support for a group-based approach. However, GLA:D or other group-based physical therapy models have not been widely implemented in the US. We recently completed a randomized clinical trial comparing group-based PT with traditional individual PT among Veterans with knee OA at the Durham VA Healthcare System (DVAHCS) [6]. Both group and individual PT interventions for that trial were based on recommended components of PT for knee OA in clinical guidelines and prior research on exercise-based interventions for knee OA [7]. In that study, participants who received either group or individual PT improved in pain and functional outcomes, with no difference in these outcomes between the two treatment delivery models. This is important since the group-based PT model required fewer clinician hours per patient to deliver, and therefore has the potential to improve operational efficiency and generate healthcare savings while maintaining comparable patient-level outcomes. Based on results of our previously conducted trial and positive experiences expressed by both staff and participants regarding the group-based PT program for Veterans with knee OA, the Physical Medicine and Rehabilitation Service at the DVAHCS began offering this program as a regular clinical service in October 2015. It is important to evaluate implementation of new clinical programs, since fidelity, effectiveness and other metrics may differ from intervention delivery in the context of the controlled research setting. As part of a broader project, the Optimizing Function and Independence Quality Enhancement Research Initiative program [8], we conducted a one-year evaluation of the group-based PT program for knee OA (“Group PT”) at the DVAHCS beginning August 1, 2016. Specifically, we evaluated both implementation metrics (e.g., penetration and fidelity) and clinical outcomes (e.g., changes in pain and function) of Group PT.

## Methods

This project was reviewed by the DVAHCS Institutional Review Board and determined to be a non-research operations activity as described in Veterans Health Administration Handbook 1058.05 (November 11, 2016). The authors followed the Revised Standards for Quality Improvement Reporting Excellence (SQUIRE 2.0) checklist in the development of this manuscript.

### Group PT for knee OA program

Veterans at the DVAHCS with a diagnosis of knee OA are eligible for this program. Veterans are referred to Group PT via an electronic referral from a treating health care provider, including any primary care or specialty medical provider or physical therapists on staff (i.e., if evaluation in response to a PT consult indicates Group PT may be appropriate). The referral specifies inclusion criteria of 1) Knee OA diagnosed by imaging, 2) Cognitively intact, 3) No history of falls, 4) No neurological deficits (i.e. post-stroke, Parkinson’s, multiple sclerosis), and 5) Patient >50 years of age. The referral also notes, “This consult is NOT for patients complaining of knee pain without imaging changes”. Once the referral is issued, it is then triaged by the physical therapist to determine if the Veteran meets inclusion criteria. Veterans are then contacted via telephone by a scheduler within Physical Medicine and Rehab Service (PM&RS) and can agree to be scheduled for an initial session in the Group PT program, request individual PT instead, or decline PT all together; reasons for declining are documented within the electronic health record (EHR). At the initial Group PT session, the physical therapist performs an assessment, collects baseline measures (described below), and determines whether the patient is appropriate for continuing to participate in the Group PT program. In some cases the physical therapist may decide, based on clinical judgement, that individual PT is more appropriate for a given patient due to comorbid physical health conditions (e.g., severe medical conditions that limit activity or require closer supervision) or psychological health conditions that are exacerbated by group situations. This determination can be made on the basis of medical record review when the patient is referred or following initial in-person evaluation in the Group PT program. Additionally, Veterans referred to PT for knee pain via standard consult process may subsequently be referred to the class if deemed appropriate by the evaluating PT.

For each participant, the Group PT program includes 6 one-hour sessions. Although participants are encouraged to attend sessions on consecutive weeks, sessions can be rescheduled when there are conflicts. One physical therapist leads each Group PT session with a maximum of 10 Veterans per class. Veteran program admission occurs on a rolling basis, as space allows. Group sessions are modeled after those developed and tested in the previous clinical trial [6], where the physical therapist engages attendees in group discussion or instruction (approximately 10 min) regarding exercise successes and barriers (3 sessions), overview of exercise and knee OA (1 session), joint protection (1 session) and activity pacing (1 session). The remainder of the session involves group stretching exercises (including seated hamstring stretch, standing quadriceps stretch, and standing calf stretch) and strengthening exercises (including standing calf raises, single leg balance, squats or min-squats, step-ups, partial stands from a chair and seated hip abduction with a therapy band). Participants leave each session with handout instructions for home exercise and a therapy band for at-home resistance exercises. Participants are encouraged to stretch daily and perform strengthening exercise three times per week, beginning with a standard set of exercises and encouraged to add repetitions, sets or resistance as they experience improvement.

### Participant characteristics

Participants’ age, sex and residential distance from the DVAHCS (in miles) were obtained from the EHR. At the first Group PT session, participants reported the number of years they had knee pain.

### Implementation metrics

Implementation metrics were assessed using EHR data stored in the VA Corporate Data Warehouse (CDW). For our evaluation, we examined a cohort of patients who were referred to the Group Physical Therapy for Knee OA sessions between August 1, 2016 and July 31, 2017. We also collected information about the service location and staff position ordering the referral from EHR data.

#### Implementation penetration-Referrals

To assess program penetration (e.g. the extent to which the service reaches the target population) we identified the total number of referrals issued during the one-year cohort entry period, as well as by clinical service that issued the referral. We also observed the number of patients who scheduled an initial Group PT session and the number who initiated the program (defined as completing at least one appointment), from among those with referrals. Reasons for not initiating Group PT were extracted from the EHR.

#### Implementation Fidelity-Attendance at Group PT Sessions

We examined several measures of program fidelity. First, we calculated the number of Group PT sessions attended by each participant who initiated Group PT. Attendance at sessions was verified via chart review of the EHR appointment data between October 1, 2016 through September 30, 2017, to allow all patients in the cohort time to complete the 6-session program. We calculated the mean and standard deviation (SD) of sessions attended per person. Second, we computed the mean number of attendees per Group PT session. Third, we computed the number and proportion of patients who completed all 6 sessions. Reasons participants did not attend all six sessions were extracted from the EHR when available or were surveyed by phone to inquire about reasons for discontinuation (attendance of < 5 sessions).

### Patient-level measures (impact)

We assessed physical function through patient self-reported and PT evaluation of physical function at the beginning of their first session and at the end of the last (sixth) session. Satisfaction was measured after their last session only.

#### Western Ontario and McMaster universities osteoarthritis index (WOMAC)

The WOMAC is a measure of lower extremity pain (5 items), stiffness (2 items), and function (17 items) [9, 10]. All items were rated on a Likert scale of 0 (no symptoms) to 4 (extreme symptoms), with a total range of 0–96 and higher scores indicating worse symptoms. Pain and function subscales were also examined separately, with ranges of 0–20 and 0–68, respectively.

#### Pain intensity

Participants reported their average daily level of pain on a numeric scale of 0 (no pain) to 10 (worst pain).

#### 30 –second chair rise

Participants began in a seated position and were instructed to stand up completely and return to the seated position, without using their arms or hands for support, as many times as they could during a 30-s period [11]. Participants were given a score of 0 if they could not complete at least one chair rise.

#### Single leg stand

Participants were instructed to stand on one leg, with eyes open and without holding onto anything for support, for as long as they could [12, 13]. This was repeated for both legs, with a maximum time of 60 s each. Participants were given a score of 0 if they could not complete the test.

#### Patient feedback on the group PT program

Participants indicated their overall satisfaction with the Group PT program as: not satisfied, somewhat unsatisfied, neutral, somewhat satisfied, and completely satisfied. Participants were asked how they would rate their ability to deal with daily problems with knee function and pain after completing the program, compared with before the program, with response options of: much worse, a little worse, about the same, a little better and much better. Finally, participants were asked whether they would recommend the Group PT program to other Veterans if they needed VA physical therapy for a condition such as theirs, with response options of: definitely no, probably no, probably yes and definitely yes.

## Results

### Participant characteristics

Table 1 shows characteristics of patients with a referral for Group PT, as well as those who attended ≥1 session and ≥ 5 sessions. There were no substantial differences in patient characteristics across these groups. Among patients attending ≥1 Group PT session (*n* = 71), 89% were men and the mean age was 62.1 (standard deviation (SD) = 11.2). These participants lived an average of 24.5 miles (SD = 26.5) from DVAHCS and reported an average duration of 9.64 years (SD = 10.0) of knee pain during their first Group PT session.

**Table 1:** Patient Characteristics Based on Referral and Attendance Status

	All Patients Referred (*N* = 152)	Patients Attending ≥1 Group PT Session (*N* = 71)	Patients Attending ≥5 Group PT Sessions (*N* = 49)
% Male	89%	89%	88%
Mean Age, years (SD)	62.1 (11.2)	64.0 (9.8)	65.5 (9.8)
Mean Distance Lived from DVAHCS, miles (SD)	34.3 (45.5)	24.5 (26.5)	30.9 (44.3)
Mean Total WOMAC Score (SD)	–	56.9 (18.8)	56.8 (15.8)
Mean Knee Pain Score, 0–10 (SD)	–	6.4 (2.0)	6.7 (1.9)

### Implementation metrics

#### Referrals

**Fig. 1:**
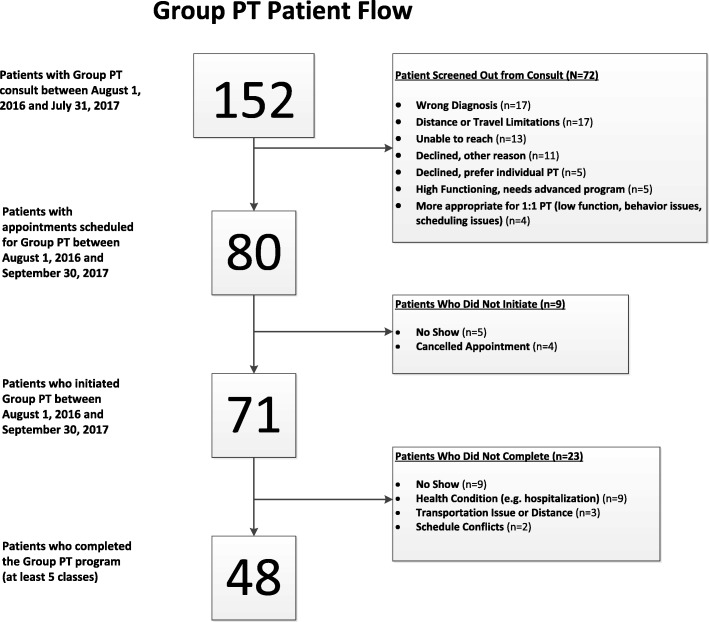
Participant Study Flow

**Fig. 2:**
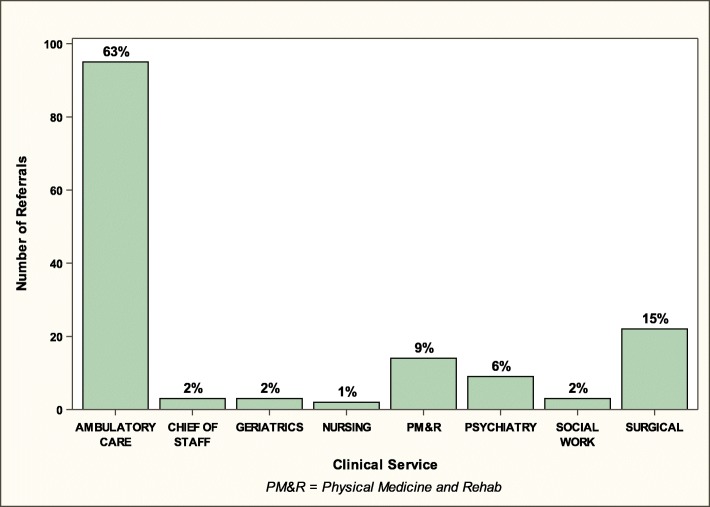
Referrals by Clinical Service (N and %)

During the one-year period, 152 patients had a referral issued for the Group PT program (Fig. 1). Referrals were issued by 8 different clinical services at the DVAHCS, with the majority from the Ambulatory Care Service (Fig. 2). Among the 152 patients with a referral, 80 (53%) had an appointment scheduled. For the 72 with no Group PT appointment scheduled, multiple reasons for not initiating Group PT could be recorded in the EHR for each patient. The most common reasons were: patient was determined by the physical therapist to be inappropriate for the program (*n* = 44), patient did not want to participate in a group program (*n* = 15), or scheduler could not reach the patient (*n* = 13) (Fig. 1).

#### Attendance

Among patients with at least one scheduled Group PT session (*n* = 80), 71 attended at least 1 Group PT session. The mean number of attended sessions (out of a total possible of 6) was 4.1 (SD = 2.4) and 61.3% (*n* = 49) attended at least 5 sessions. For the majority of patients who did not attend at least 5 Group PT sessions (*n* = 14, 45%), no specific reason was recorded in the EHR (e.g., the patient was a “no show” and did not respond or return to the session); among the reasons listed health problems (e.g. hospitalization) were the most common (Fig. 1). The mean number of attendees per session was 7.1 (SD = 2.8).

### Patient-level measures

**Fig. 3:**
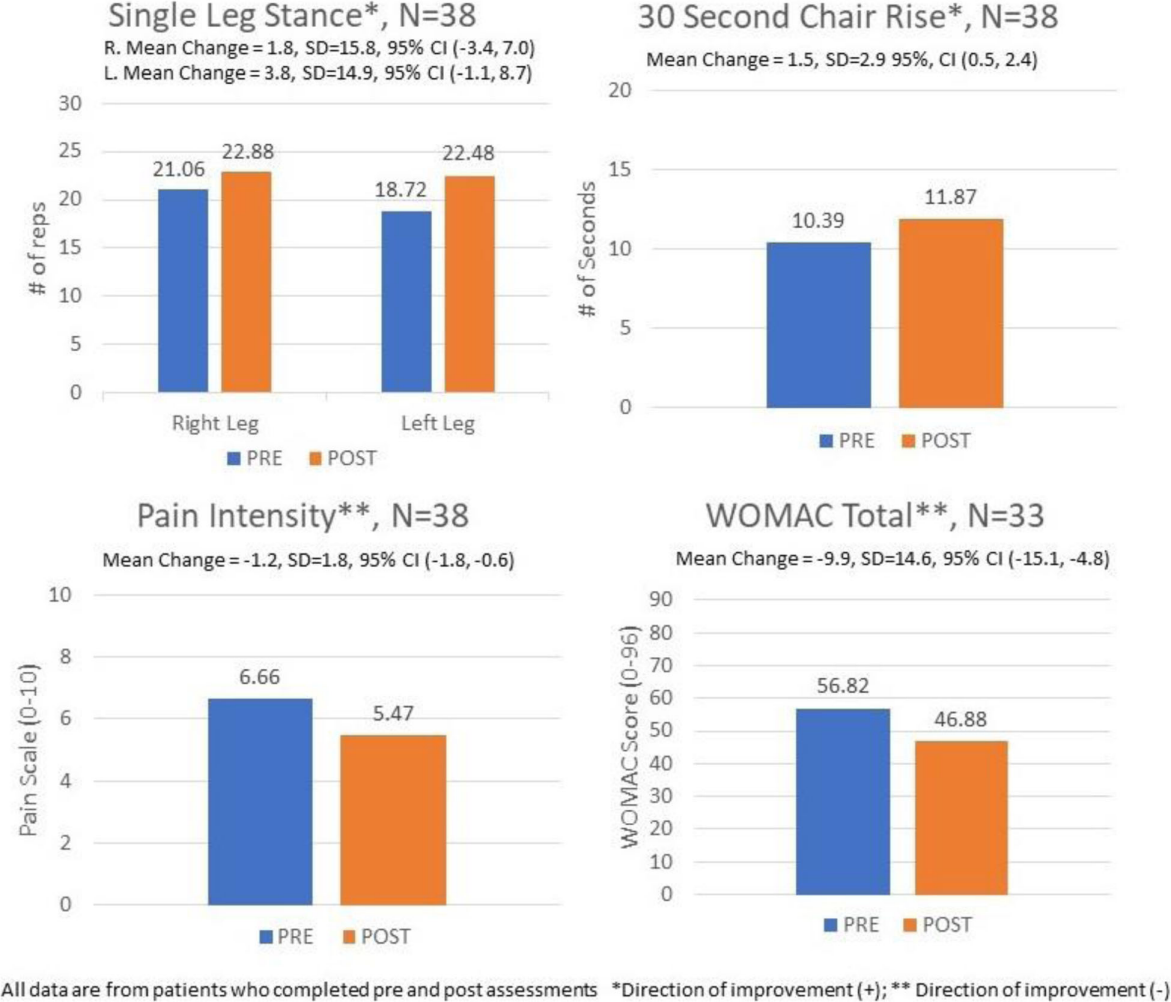
Function and Pain Measures Before and After the Group PT Program

A total of 69 patients reported physical function measures at baseline (first session), and 38 reported at the end of the Group PT program. The main reason for this loss to follow-up was that several patients did not attend their last scheduled session. Figure 3 displays patient-level measures for those who completed both baseline and follow-up. The appendix shows data for all patients who initiated the Group PT program.

#### WOMAC

Among 38 participants who completed both baseline and follow-up Group PT WOMAC surveys, the mean WOMAC score declined from 56.8 (SD = 15.8) to 46.9 (SD = 14.0), representing a 17% improvement. The mean WOMAC function score improved from 39.0 (SD = 11.8) to 32.5 (SD = 10.8), and the mean WOMAC pain score improved from 11.6 (SD = 3.3) to 9.5 (SD = 3.2) (see Table 2 in Appendix).

#### Pain intensity

Among 38 participants who completed the pain intensity measure at both baseline and follow-up, the mean score improved from 6.7 (SD = 1.9) to 5.6 (SD = 1.9).

#### 30 –second chair rise

Among 38 participants who completed this test at both baseline and follow-up, the mean number of chair rises completed increased from 10.4 (SD = 5.1) to 11.9 (SD = 5.0).

#### Single leg stand

Among 38 participants who completed this test at both baseline and follow-up, stand time for the right leg remained approximately the same: 21.1 s (SD = 21.4) at baseline and 22.9 s (SD = 22.8) at follow-up. For the left leg, stand time increased from 18.7 s (SD = 21.5) to 22.5 s (SD = 23.2).

#### Patient feedback on the group PT program

Among 33 participants who completed the satisfaction measure, 27 (82%) reported being “completely satisfied,” 5 (15%) “somewhat satisfied,” and 1 (3%) “neutral;” 0 patients were dissatisfied. Regarding participants’ ratings of ability to deal with daily problems with knee function and pain now, compared with before the Group PT program, 15 (45%) responded “much better,” 14 (42%) responded “a little better,” and 4 (12%) responded “about the same;” 0 patients responded that it was worse. Thirty participants (91%) responded that they would “definitely” recommend and 3 [9] responded that they would “probably” recommend Group PT to other Veterans in need of VA physical therapy for a condition like theirs.

## Discussion

This quality improvement project evaluated the roll-out of a Group PT program for Veterans with knee OA and found that this service is feasible to deliver and associated with improved OA-related outcomes and high satisfaction. These results concur with findings of dissemination of GLA:D, which has also been associated with improved pain and function in registry-based analyses of enrolled patients [5]. The GLA:D patient program includes 2 education and 12 neuromuscular sessions conducted twice per week over 6 weeks. Our Group PT program requires fewer visits than GLA:D and therefore offers a different option that may be useful when it is not feasible for patients to attend twice weekly sessions.

### Implementation metrics

Health care providers at the DVAHCS referred about 150 patients to the Group PT program during the one-year evaluation phase, and these referrals came from providers in a variety of services. While this referral rate illustrates interest in the clinical program by providers, there is still a need to increase the rate of referrals, highlighting the importance of communication across services about the availability of new programs and processes for referral. Because the vast majority of patients with knee OA are treated in primary care (vs. smaller proportions seen by specialty services), we believe that augmenting referrals by primary care providers will be important for increasing use of this program. Our findings also reveal that a relatively high proportion of referred patients did not initiate the Group PT program (Fig. 1). In some cases (*N* = 16) this was because the patient did not have a knee OA diagnosis; this occurred even though eligibility criteria for the program were listed in the EHR consult and illustrates that ongoing education of referring providers may be required to optimize appropriate referrals. There were also patients who could not participate because they lived too far away from the DVAHCS or had travel limitations. It is important for providers to inform patients of the nature and requirements of a program and assess interest prior to making a referral, which may substantially enhance the efficiency of the referral process and ensure that patients are connected with the most appropriate treatment option quickly.

Participant attendance at Group PT sessions was moderate, with a mean of 4 sessions attended and 32% completing at least 5 sessions out of 6. This is similar to the attendance rate observed in the randomized trial of the Group PT for knee OA program, illustrating the feasibility of implementing this service outside of the controlled research context, in which participants were called by research personnel to remind them of Group PT sessions [6]. Attendance can be a challenge with programs that require multiple in-person sessions, a barrier that is further compounded for those who travel relatively long distances to attend sessions. During this evaluation, the average distance traveled by those who initiated the program was roughly 25 miles. Sites considering a program like this should consider the likely catchment area and whether other types of supports, such as mobile health tools, may be able to augment in-person sessions, potentially requiring fewer visits.

### Patient measures

Participants who completed the Group PT program experienced improvements in multiple measures of pain and function. Total WOMAC scores improved 17%, which is a clinically meaningful improvement in the context of this type of intervention [14]. In addition, this is a similar percent improvement in total WOMAC score as was observed in our randomized clinical trial of Group PT for knee OA [6], suggesting that the benefits of this program extend into clinical settings outside of a controlled research context. The mean 1.2-point improvement on the pain intensity scale also meets the threshold for a clinically meaningful difference [15]. The mean increase of about 1.5 chair stands in 30 s is also a clinically relevant improvement [16]. For these participants’, mean stand time improved somewhat in both the right and left legs (1.8 and 3.8 s, respectively), though the confidence intervals for these changes were relatively wide and crossed 0. In addition to improvements in patient-reported symptoms and some objectively assessed function measures, participants expressed a high degree of satisfaction with the program, with the vast majority being “completely satisfied.” This is also an important finding, since the group-based model differs from patients’ typical experiences of PT care, with less individual attention.

### Limitations

This evaluation was conducted at a single VA and involved a small, mostly male sample; all of these factors can limit the generalizability. In addition, there may be differences in characteristics of patients who initiated Group PT compared with those who were referred but did not initiate, as well as between those who attended vs. did not attend all Group PT sessions. Among 49 patients who completed at least 5 Group PT sessions, follow-up outcomes could only be obtained from 38, primarily because many of these individuals did not attend their last planned session, when the follow-up data were collected. Although assessment of physical function was included, only 2 specific tests were administered due to time constraints in the context of this clinically-based (non-research) setting. A more comprehensive set of function measures, as well as measures such as physical activity and quality of life, would be useful for further evaluating program effectiveness, along with longer-term follow-up. Finally, it was not feasible to determine changes in the proportion of patients with knee OA who received individual PT at DVAHCS before vs. after the Group PT program was implemented. This is because knee OA is not coded consistently during clinic visits, and therefore the total number of patients at DVAHCS with knee OA diagnoses during a particular time frame could not be ascertained. Understanding the impact of Group PT implementation on use of individual PT services is an important future direction for this research, and would require systems to accurately document knee OA diagnoses in both individual and group clinic settings.

## Conclusion

Overall this quality improvement project demonstrated that the Group PT program that was developed and tested in a controlled clinical trial can result in clinically meaningful improvements when implemented in a real-world clinical setting. The number of provider referrals and high patient satisfaction ratings support the value of the Group PT program from both provider and patient perspectives. These findings are important because a group-based model for delivering PT can improve efficiency and access; this may be particularly beneficial for low-resource settings and address issues of physical therapist shortages in some health systems. Patient attendance can be a challenge for clinical services like Group PT that require multiple in-person visits, and our data indicate this may be a particular barrier for patients who live further distances from their health care facility. To maximize the efficiency and effectiveness of a Group PT program, health care systems should consider initial screening to assess patient interest and engagement, as well as strategies to foster attendance among patients who initiate. In addition, the reach of this type of program to rural-dwelling patients could be expanded through conducting Group PT sessions at community-based outpatient clinics or through telemedicine-based visits or mobile health tools to enhance delivery of the intervention.

## Data Availability

The data sets generated during the current study are not publicly available because the include personally identifiable data from study participants. However, deidentified data are available from the corresponding author on reasonable request.

## Appendix

**Table 2:** Patient Level Measures

Measure	Pre-Group PT Program		Post-Group PT Program	
	N	Mean (SD)	N	Mean (SD)
30 Second Chair Rise	69	10.5 (4.7)	38	11.9 (5.0)
Single Leg Stance (Right)	69	20.4 (22.9)	38	22.9 (22.8)
Single Leg Stance (Left)	69	18.0 (21.7)	38	22.5 (23.2)
Pain Scale (0–10)	69	6.4 (2.0)	38	5.5 (1.9)
WOMAC Total (Out of 96)	66	56.9 (18.8)	33	46.9 (14.0)
WOMAC Function Subscale	65	39.8 (14.1)	32	32.5 (10.8)
WOMAC Pain Subscale	65	11.6 (3.9)	32	9.5 (3.2)
WOMAC Stiffness Subscale	65	5.4 (2.0)	32	4.5 (1.5)

*WOMAC* Western Ontario and McMaster Universities Osteoarthritis Index

## Abbreviations

CDW: Corporate Data Warehouse
DVAHCS: Durham VA Healthcare System
EHR: Electronic Health Record
OA: Osteoarthritis
PM & RS: Physical Medicine and Rehabilitation Service
PT: Physical Therapy
SD: Standard Deviation
SQUIRE: Standards for Quality Improvement Reporting Excellence
VA: Veterans Administration
WOMAC: Western Ontario and McMaster Universities Osteoarthritis Index
